# Current GBIF occurrence data demonstrates both promise and limitations for potential red listing of spiders

**DOI:** 10.3897/BDJ.7.e47369

**Published:** 2019-12-19

**Authors:** Vaughn Shirey, Sini Seppälä, Vasco Veiga Branco, Pedro Cardoso

**Affiliations:** 1 Laboratory for Integrative Biodiversity Research (LIBRe), Finnish Museum of Natural History, University of Helsinki, Helsinki, Finland Laboratory for Integrative Biodiversity Research (LIBRe), Finnish Museum of Natural History, University of Helsinki Helsinki Finland; 2 Georgetown University, Washington, DC, United States of America Georgetown University Washington, DC United States of America; 3 The Academy of Natural Sciences of Drexel University, Philadelphia, United States of America The Academy of Natural Sciences of Drexel University Philadelphia United States of America

**Keywords:** Araneae, arthropoda, conservation, extent of occurrence, IUCN

## Abstract

Conservation assessments of hyperdiverse groups of organisms are often challenging and limited by the availability of occurrence data needed to calculate assessment metrics such as extent of occurrence (EOO). Spiders represent one such diverse group and have historically been assessed using primary literature with retrospective georeferencing. Here we demonstrate the differences in estimations of EOO and hypothetical IUCN Red List classifications for two extensive spider datasets comprising 479 species in total. The EOO were estimated and compared using literature-based assessments, Global Biodiversity Information Facility (GBIF)-based assessments and combined data assessments. We found that although few changes to hypothetical IUCN Red List classifications occurred with the addition of GBIF data, some species (3.3%) which could previously not be classified could now be assessed with the addition of GBIF data. In addition, the hypothetical classification changed for others (1.5%). On the other hand, GBIF data alone did not provide enough data for 88.7% of species. These results demonstrate the potential of GBIF data to serve as an additional source of information for conservation assessments, complementing literature data, but not particularly useful on its own as it stands right now for spiders.

## Introduction

The mobilisation of biodiversity data through aggregating platforms such as the Global Biodiversity Information Facility (GBIF) has generated excitement about the potential for applying such publicly available data towards filling gaps in biological knowledge (Edwards 2004). To this end, the ability to predict species distributions more accurately using aggregated occurrence data may have broad implications for land management, environmental policy, ecosystem monitoring and conservation. Examples of such potential include the utilisation of GBIF occurrence data towards prioritising areas and species for conservation purposes (Alburquerque and Beier 2016, Miličić et al. 2017).

For many taxa, conservation assessments are conducted through the International Union for Conservation of Nature’s (IUCN) Red List framework, which provides information about species threat levels. The Red List also aims to monitor global trends in biodiversity and inform policy-makers on the conservation of nature (IUCN 2012). Given the scarcity of data on population numbers and trends for megadiverse taxa, these assessments are mostly based on the geographical range of a taxon and how that range has changed over time. Thus, a comprehensive, or at least representative, set of georeferenced occurrence data is needed to assess the potential threat to a species.

Araneae represent one group of largely understudied and under-sampled organisms, still lagging other taxa in terms of representative data in GBIF (Troudet et al. 2017). In addition to this lack of occurrence data, additional knowledge gaps in the ability to conserve spiders and other arthropods are apparent, including taxon-specific deficiencies in ecological and biogeographic knowledge (Cardoso et al. 2011). With acknowledgement of this deficiency in spider data, assessing the utility of our current knowledge base is essential for promoting further digitisation and discovery of species, their natural history and eventual conservation. In this study, we aim to test the utility of current GBIF occurrence data in the extinction risk assessment of spiders using two large-scale assessments as examples.

## Material and methods

Two extensive datasets were used to assess the applicability of GBIF occurrence data in threat assessments. The first consists of a random selection of 200 species from the World Spider Catalog (Natural History Museum Bern 2017), a global database of all recognised species names for spiders. These were chosen for another study concerning a baseline spider Sampled Red List Index - SRLI (Seppälä et al. 2018a, Seppälä et al. 2018b, Seppälä et al. 2018c, Seppälä et al. 2018d). The SRLI is a methodological approach to quantify global trends towards extinction of speciose taxa (Baillie et al. 2008, Lewis and Senior 2011) and these data will contribute to its inception for spiders. Occurrence data for these 200 species were collected from the taxonomic bibliography available at the same database until the end of 2017. Data from publications found on Google Scholar and several other online sources were also included in this dataset and detailed in Cardoso et al. (2019). The use of literature as the baseline for these assessments follows established IUCN procedures for Red List methodologies (IUCN 2012).

The second dataset was compiled for all 279 endemic spider species of the Iberian Peninsula (Continental Portugal, Spain, Andorra and Gibraltar, plus the Balearic Islands), collected from a bibliographic database on species occurrences in the region (Branco et al. 2019). This database includes all published citations until the end of 2018.

Geographic coordinates were obtained for each locality across both datasets using literature sources and georeferenced locality data. To these data, we added all georeferenced records from GBIF of the same 200 (see original data references to GBIF in Seppälä et al. 2018a, Seppälä et al. 2018b, Seppälä et al. 2018c, Seppälä et al. 2018d) plus 279 species (GBIF.org 2019). Non-georeferenced data were removed from the analysis as they cannot be utilised in our spatial metric calculation, species lacking any georeferenced data being designated as Data Deficient (DD). Our goal was to use GBIF data without any further modification and/or annotation and, therefore, we did not georeference records lacking coordinates. Coordinates obtained from GBIF were reviewed alongside known species distributions to determine if dubious localities existed (e.g. records of Iberian endemics occurring outside of their known ranges). We found no records that warranted deletion from dubious localities.

Our analysis consisted of comparing IUCN classifications assigned to each species by using the GBIF, literature and combined literature and GBIF datasets in an Extent of Occurrence (EOO) calculation. EOO is defined as the area contained within the shortest continuous imaginary boundary that can be drawn to encompass all records (IUCN 2012). Note that to build a minimum convex polygon, at least three data points are needed, otherwise the species was classified as Data Deficient. Particular EOO thresholds must be met in order for a species to be considered Critically Endangered (CR, < 100 km^2^), Endangered (EN, < 5,000 km^2^), Vulnerable (VU, < 20,000 km^2^) or Near Threatened (NT, < 30,000 km^2^). Species with no calculated area are classified as Data Deficient (DD). Although other criteria must be met for a full IUCN assessment, we did not consider them here in the context of spatial occurrence data. EOO was calculated by using the R-package “red” (v.1.4.0) (Cardoso 2018) in R version 3.6.0 (R Core Team 2019).

R scripts used for data retrieval and processing are available on GitHub (https://github.com/vmshirey/spiders) where the dated version of this repository that corresponds to this publication is December 2019. The literature datasets were contributed to GBIF and consisted of 2,378 records for the global list and 30,141 records for all the Iberian taxa (Cezón and Cardoso 2019, Cardoso et al. 2019).

## Results

**Global Spider Taxa**

Using GBIF data alone, 17.5% of species from our global taxon list could be classified into a hypothetical IUCN category. A total of 40.0% could be classified using literature data alone and 45.5% could be classified using the combined GBIF and literature datasets (Table 1). With the addition of GBIF data to the literature dataset, 6.5% of species shifted their classification. A few species, in particular, suffered considerable downgrades in their hypothetical classification, namely *Myrmarachne bicolor* (L. Koch, 1879) (VU to LC). This change was due to an addition of 16 GBIF records to the literature dataset of 7 records.

**Iberian Endemic Spider Taxa**

Using GBIF data alone, 6.8% of Iberian endemic species could be classified into a hypothetical IUCN category. A total of 58.1% could be classified using literature data alone and 59.9% could be classified using the combined GBIF and literature datasets (Table 2). With the addition of GBIF data to the literature dataset, 4.7% of records shifted classification. A few species, in particular, suffered considerable downgrades in their hypothetical classification, including *Micrommata aragonensis* Urones, 2004 and *M. aljibica* Urones, 2004 (both EN to LC). These changes were due to the addition of 1 and 2 GBIF records to the literature dataset of 3 and 4 records, respectively.

**Overall Summary**

Overall, we found that, although few changes to hypothetical IUCN Red List classifications occurred with the addition of GBIF data, some species (3.3%), which could previously not be classified, could now be assessed with the addition of GBIF data. In addition, the hypothetical classification changed for others (1.5%). On the other hand, GBIF data alone did not provide enough data for 88.7% of species.

## Discussion

The status of current GBIF data for extinction risk assessment of spiders shows both promise and limitations. These results largely fall in line with prior exploration of GBIF data in species conservation assessments, including the need for experts in taxonomy to review the validity of records and taxonomic determinations (Hjarding et al. 2014). Recent analyses of museum datasets have suggested that researchers take a critical lens to using museum occurrence data, as taxonomic misidentification and spatial biases are known to occur (Nekola et al. 2019). In addition, particular research disciplines may focus on collecting and digitising specimens related to taxonomic work that could influence over- and undersampling of particular species. These pitfalls are difficult to mitigate when utilising online data without validation of species taxonomy or correct label transcriptions. Thus, results presented using such data (and in particular, results in which a few records drastically change results) should not be taken as absolute fact. Yet, in a few cases, GBIF data might contribute more records without expanding species occurrence ranges if the new records fall inside the polygon encompassed by the old ones (Beck et al. 2013).

Despite this, promising results in our study include the change of hypothetical EOO-based classification amongst species listed as threatened across both species lists. Moreover, any change of risk assessment classifications from Data Deficient (DD) is notable. These changes provide initial assessments to previously DD taxa, which may add up to very large proportions of assessments on many hyperdiverse groups, including spiders (Seppälä et al. 2018d). Additionally, other researchers have focused on using GBIF data to partly automate the process of Red Listing, including the calculation of spatial metrics (Bachman et al. 2011, https://spbachman.shinyapps.io/rapidLC/).

Although such advancements should be noted, it is worth realising that just 6.5% and 4.7% of the taxa in the global and Iberian datasets, respectively, change their hypothetical IUCN classifications. The low rates of observed classification shift could be an artifact of the aforementioned data pitfalls for spiders in GBIF, which strengthens the argument for more collection, observation and/or digitisation of data. Retrospective georeferencing of locality data within GBIF will also serve to further enhance these metrics. Currently (as of December 2019), 93% of GBIF records are georeferenced; however, coordinates are less often available for certain groups, such as Araneae (88%).

Despite current limitations, we believe that there is potential for the use of GBIF occurrence data in Red List assessments. Additional data sourced from GBIF will help refine IUCN spatial metrics, in particular EOO, even when considering the currently identified pitfalls of GBIF data. While these metrics should, in general, not be calculated with GBIF data alone, it is important to consider GBIF as a source of additional information. Moreover, the addition of more data from collections and community-based observations improves the potential applicability of GBIF data in Red List classification assessments.

## Figures and Tables

**Table 1.** Hypothetical IUCN Red List classifications for the global spider list.

	Literature	GBIF	Combined
DD	120	165	109
CR	3	2	6
EN	10	3	10
VU	4	0	6
NT	3	0	3
LC	60	30	66

**Table 2.** Hypothetical IUCN Red List classifications for Iberian endemics by data source.

	Literature	GBIF	Combined
DD	117	260	112
CR	17	4	16
EN	53	7	55
VU	29	3	28
NT	5	0	7
LC	58	5	61
